# Utilization of at-home tests for coronavirus disease 2019 (COVID-19) among healthcare workers in Chicago

**DOI:** 10.1017/ash.2024.17

**Published:** 2024-04-24

**Authors:** Nathaly Valdivia, Lisa R. Hirschhorn, Thanh-Huyen Vu, Cerina Dubois, Judith T. Moskowitz, John T. Wilkins, Charlesnika T. Evans

**Affiliations:** 1 Center for Health Services and Outcomes Research, Institute for Public Health and Medicine, Feinberg School of Medicine, Northwestern University, Chicago, IL, USA; 2 Department of Medical Social Sciences, Feinberg School of Medicine, Northwestern University, Chicago, IL, USA; 3 Department of Preventive Medicine, Feinberg School of Medicine, Northwestern University, Chicago, IL, USA; 4 Institute for Public Health and Medicine, Center for Education in Health Sciences, Northwestern University, Chicago, IL, USA; 5 Division of Cardiology, Department of Medicine, Feinberg School of Medicine, Northwestern University, Chicago, IL, USA; 6 Center of Innovation for Complex Chronic Healthcare, Department of Veterans’ Affairs, Edward Hines, Jr, VA Hospital, Hines, IL, USA

## Abstract

**Objective:**

To describe utilization of at-home coronavirus disease 2019 (COVID-19) testing among healthcare workers (HCW).

**Design:**

Serial cross-sectional study.

**Setting and participants:**

HCWs in the Chicago area.

**Methods:**

Serial surveys were conducted from the Northwestern Medicine (NM HCW SARS-CoV-2) Serology Cohort Study. In April 2022, participants reflected on the past 30 days to complete an online survey regarding COVID-19 home testing. Surveys were repeated in June and November 2022. The percentage of completed home tests and ever-positive tests were reported. Multivariable Poisson regression was used to calculate prevalence rate ratios (PRR) and univariate analysis was used for association between participant characteristics with home testing and positivity.

**Results:**

Overall, 2,226 (62.4%) of 3,569 responded to the survey in April. Home testing was reported by 26.6% of respondents and 5.9% reported having at least one positive home test. Testing was highest among those 30–39 years old (35.9%) and nurses (28.3%). A positive test was associated (*P* < .001) with exposure to people, other than patients with known or suspected COVID-19. Home testing increased in June to 36.4% (positivity 19.9%) and decreased to 25% (positivity 13.5%) by November.

**Conclusion:**

Our cohort findings show the overall increase in both home testing and ever positivity from April to November – a period where changes in variants of concern of SARS-CoV-2 were reported nationwide. Having an exposure to people, other than patients with known or suspected COVID-19 was significantly associated with both, higher home testing frequency and ever-test positivity.

With healthcare workers (HCWs) being ten times more likely to be infected with COVID-19,^1^ exposure to people with COVID-19 infection is a strong factor associated with COVID-19 infection among this population.^2^ While mandated COVID-19 vaccines have proven effective in protection against severe COVID-19 infection,^3^ risk for infection and reinfection in HCWs persist due to ongoing exposures, waning immunity, and emerging COVID-19 variants.^4^ Frequent testing and reporting may help reduce transmission in healthcare settings.^5^

At-home rapid COVID-19 antigen tests were made widely available and free in the U.S., by the Biden administration in early 2022.^6^ Northwestern Medicine, a large healthcare system in Illinois, also provided a limited supply of free home tests to students starting in March 2022.^7^ Northwestern faculty, staff, and students were provided with free masks and free COVID-19 in-person testing (reverse-transcription polymerase chain reaction (RT-PCR) and rapid antigen) at any Northwestern location. Unlike the free home test distribution to students, home tests for faculty and staff were advertised as being free through the federal government program and with most insurances.^8^

Despite the fact that HCWs have access to testing options at their workplace, it is important to understand the utilization of home tests, because home testing as a molecular testing alternative is an additional strategy that can be employed to reduce the spread of SARS-CoV-2 in healthcare settings. A recent study from the United Kingdom reported that weekly screenings, regardless of presenting symptoms, among healthcare workers is estimated to reduce SARS-CoV-2 transmission by 23%.^9^ Adding a further layer of protection for HCWs, like providing home testing kits, allows HCWs to rely on the self-administered home test result prior to entering a healthcare facility and further reducing the spread of SARS-CoV-2.

Using survey data from the established Northwestern Medicine (NM) HCW SARS-CoV-2 Serology Cohort Study,^10^ we conducted an exploratory analysis of self-reported COVID-19 home testing to assess occupational predictors, positivity rates and what perceived exposures were associated with home testing among HCWs.

## Methods

### Study design and sample

This exploratory cross-sectional analysis gathered information from an ongoing cohort study of NM HCWs practicing in the Chicagoland area and surrounding suburbs.^10,11^ Serial surveys have been conducted since May 2020. Questions about home testing were added in April 2022. A total of 3,569 HCWs were invited to participate in the survey in April 2022. This analysis focuses on HCWs who consented to continue in the cohort study and completed the online survey in April. Subsequent surveys from June and November 2022 were analyzed for comparison to April to assess how home testing changed over these time periods.

### Survey measures

During baseline recruitment, participants reported on demographics, including age, gender, race and ethnicity, occupation, and comorbidities. For this analysis, participant age was categorized into four groups (18–29, 30–39, 40–49, and over 50), occupation into four groups (administrative role, nurse, physician, and other), and a composite comorbidity variable included if a HCW reported any one of the following conditions: cancer, hypertension, immunocompromised, liver disease, or diabetes.^12^

The home testing question asked whether the HCW completed a home test in the past 30 days. An answer of yes then prompted further questions asking: how many tests were conducted and of those how many were positive. Participants were also asked about their suspected COVID-19 exposure encounters within the past 30 days, regardless of whether they took a COVID-19 test or not. If they reported an exposure in the past 30 days, participants were asked the nature of the exposure: patients with known or suspected COVID-19 and/or having an exposure to people, other than patients with known or suspected COVID-19.

### Statistical analysis

Chi-square statistical tests were used for comparison analysis of the categorical predictor variables and their association with our dichotomous outcome variables: completion of a home test and ever-test positivity. We conducted an unadjusted univariate analysis which included variable frequencies, standard deviations (SD) for means (M), and *P*-values. Significant associations were classified as having a *P*-value ≤ .05. Significant unadjusted variables were used to guide the selection of variables for our adjusted model using Poisson regression analysis. One model analyzed the association between HCW demographics and suspected exposures within the past 30 days, by conducting an at-home test. Only those that remained significant or if their presence changed the model results, due to possible confounding, were kept in the final model. The second model was to assess the same factors with ever having a positive test. Prevalence rate ratios (PRR) and 95% confidence intervals (CI) were reported for the adjusted models. All analyses were conducted using SAS (Statistical Analysis Software) OnDemand for Academics. This study was approved by the Northwestern University Institutional Review Board, and all participants gave written informed consent.

## Results

### Home testing in April 2022

A total of 2,226 of the 3,569 (62.4%) HCWs responded to the online survey and answered the question regarding home testing in April 2022. When compared to non-respondents, HCWs that responded to the survey were 14% less likely to be nurses, 10% less likely to be non-Hispanic white race, and they were slightly older.

Table 1 contains participant demographics, occupation, comorbidities, and exposure concerns of the past 30 days from the time they took the survey. One quarter (26.6%) of respondents completed a home test. 35.9% of HCWs between the ages of 30 and 39 reported higher testing frequencies compared to younger and older HCWs (*P* < .001). Completing a home test varied by occupation (*P* < .001), 28.3% of nurses, followed by 25.6% of physicians. HCWs without known comorbidities reported a higher frequency of completing a home test, compared to HCWs with known comorbidities (76.7% vs 23.4%, *P* < .001). About a quarter of HCWs that completed a home test, reported that within the past 30 days, they were exposed to people, other than patients with known or suspected COVID-19, as opposed to those who did not complete a home test (26.2% vs 13.4%, *P* < .001). No association was observed between home testing and reporting an exposure to patients with known or suspected COVID-19 (*P* = .13). Sex, race, and ethnicity were not associated with home testing.

**Table 1.** Participant characteristics by completion of home testing for enrolled individuals surveyed in April 2022

Characteristic	Overall, n (%)	Completed home test, n (%)		*P*-value
		Yes	No	
**n (%)**	2,226 (100.00)	591 (26.55)	1,635 (73.45)	
**Age**				**<.001**
18–29	272 (12.22)	85 (14.38)	187 (11.44)	
30–39	704 (31.63)	212 (35.87)	492 (30.09)	
40–49	539 (24.21)	149 (25.21)	390 (23.85)	
≥ 50	711 (31.94)	145 (24.53)	566 (34.62)	
**Sex**				.50
Female	1,828 (82.12)	480 (81.22)	1,348 (82.45)	
Male	398 (17.88)	111 (18.78)	287 (17.55)	
**Race/ethnicity**				.14
Asian	182 (8.18)	61 (10.32)	121 (7.40)	
Hispanic/Latino	121 (5.44)	27 (4.57)	94 (5.75)	
Non-Hispanic Black	53 (2.38)	11 (1.86)	42 (2.57)	
Non-Hispanic White	1,814 (81.49)	479 (81.05)	1,335 (81.65)	
Other/prefer not to answer^a^	56 (2.52)	13 (2.20)	43 (2.63)	
**Occupation**				**<.001**
Administrative role	315 (14.15)	82 (13.87)	233 (14.25)	
Nurse (practitioner, registered, or equivalent)	637 (28.62)	167 (28.26)	470 (28.75)	
Physician	411 (18.46)	151 (25.55)	260 (15.90)	
Other^b^	863 (38.77)	191 (32.32)	672 (41.10)	
**Comorbidities** ^c^				**<.001**
No^d^	1,523 (68.42)	453 (76.65)	1,070 (65.44)	
Yes	703 (31.58)	138 (23.35)	565 (34.56)	
**I was exposed to people, other than patients with known or suspected COVID-19**				**<.001**
No	1,219 (54.76)	275 (46.53)	944 (57.74)	
Yes	374 (16.80)	155 (26.23)	219 (13.39)	
Unsure	633 (28.44)	161 (27.24)	472 (28.87)	
**I was exposed to patients with known or suspected COVID-19**				.13
No	1,098 (49.33)	283 (47.88)	815 (49.85)	
Yes	744 (33.42)	216 (36.55)	528 (32.29)	
Unsure	384 (17.25)	92 (15.57)	292 (17.86)	

a other race comprised of HCWs identifying as multi-racial.

b other occupations: clinical coordinator, technician, environmental, food, laboratory, social worker, therapist, patient services, secretary, pastoral care, and physician/medical assistant.

c comorbidity is a composite variable comprised of HCWs having at least one of the following: cancer, hypertension, immunocompromised, liver disease, or diabetes.

d no includes didn’t answer and unsure answers.

The bold values indicate the number is statistically significant (*P* ≤ .05)

The multivariable model showed that older age groups (≥ 50 vs 18–29: PRR = 0.72, 95% CI 0.54–0.95) and those with known comorbidities (PRR = 0.76, 95% CI 0.62–0.93) were less likely to conduct home testing. Being exposed to people, other than patients with known or suspected COVID-19 was associated with home testing (PRR = 1.70, 95% CI 1.40–2.08) (Table 2).

**Table 2.** Multivariable Poisson regression analysis of association with home testing and demographic and exposure characteristics

Characteristic	Prevalence rate ratio (PRR)	95% Confidence interval (CI)	***P*** **-value**
**Age**			
18–29 (ref)	–	–	–
30–39	0.94	0.73–1.21	.65
40–49	0.89	0.68–1.17	.41
≥50	0.72	0.54–0.95	**.02**
**Occupation**			
Administrative role (ref)	–	–	–
Nurse (practitioner, registered, or equivalent)	0.96	0.73–1.25	.74
Physician	1.25	0.96–1.65	10
Other^a^	0.83	0.64–1.07	.15
**Comorbidities** ^b^			
No (ref)	–	–	–
Yes	0.76	0.62–0.93	**.007**
**I was exposed to people, other than patients with known or suspected COVID-19**			
No (ref)	–	–	–
Yes	1.70	1.40–2.08	**<.001**
Unsure	1.16	0.95–1.41	0.14

a see Table 1, footnote 2 for list of other occupations.

b see Table 1, footnote 3 for list of comorbidities.

The bold values indicate the number is statistically significant (*P* ≤ .05)

### Ever positive home tests in April 2022

Of the 591 HCWs that completed a home test in the past 30 days, from the time that they completed the survey, the average number of completed home tests was 1.83 per HCW (SD = 1.34) (Table 3). 5.9% of the 591 HCWs that completed a home test reported at least one positive test (ever positive), with the average number of a positive home tests being 1.63 per completed test (SD = 0.94). HCWs between the ages of 40 and 49 had the highest frequency of being ever positive (31.4%), however across age groups there was no statistically significant association with having a positive test (*P* = .1) (Table 3). Nurses reported the highest occurrence of ever having a positive test (40%), followed by physicians (25.7%). Reporting an exposure to people, other than patients with known or suspected COVID-19 was associated with having a positive home test compared to those without a reported exposure (77.1% vs 8.6%, *P* < .001). After looking at the independent variables, and controlling for other variables, the only significant model for ever having a positive test was the unadjusted.

**Table 3.** Participant characteristics by ever-positive home tests for April 2022

Characteristic	Overall, n (%)	Ever Positive, n (%)		*P*-value
		Yes	No	
**n (%)**	591 (100.00)	35 (5.92)	556 (94.08)	
**mean (SD)**	1.83 (1.34)	1.63 (0.94)		
**Age**				.11
18–29	85 (14.38)	9 (25.71)	76 (13.67)	
30–39	212 (35.87)	10 (28.57)	202 (36.33)	
40–49	149 (25.21)	11 (31.43)	138 (24.82)	
≥50	145 (24.53)	5 (14.29)	140 (25.18)	
**Sex**				.48
Female	480 (81.22)	30 (85.71)	450 (80.94)	
Male	111 (18.78)	5 (14.29)	106 (19.06)	
**Race/ethnicity**				.41
Asian	61 (10.32)	3 (8.57)	58 (10.43)	
Hispanic/latino	27 (4.57)	2 (5.71)	25 (4.50)	
Non-hispanic black	11 (1.86)	2 (5.71)	9 (1.62)	
Non-hispanic white	479 (81.05)	28 (80.00)	451 (81.12)	
Other/prefer not to answer^a^	13 (2.20)	0 (0.00)	13 (2.34)	
**Occupation** ^b^				.38
Administrative role	82 (13.87)	3 (8.57)	79 (14.21)	
Nurse (practitioner, registered, or equivalent)	167 (28.26)	14 (40.00)	153 (27.52)	
Physician	151 (25.55)	9 (25.71)	142 (25.54)	
Other	191 (32.32)	9 (25.71)	182 (32.73)	
**Comorbidities** ^c^				.63
No	453 (76.65)	28 (80.00)	425 (76.44)	
Yes	138 (23.35)	7 (20.00)	131 (23.56)	
**I was exposed to people, other than patients with known or suspected COVID-19**				**<.001**
No	275 (46.53)	3 (8.57)	272 (48.92)	
Yes	155 (26.23)	27 (77.14)	128 (23.02)	
Unsure	161 (27.24)	5 (14.29)	156 (28.06)	
**I was exposed to patients with known or suspected COVID-19**				.23
No	283 (47.88)	16 (45.71)	267 (48.02)	
Yes	216 (36.55)	15 (42.86)	201 (36.15)	
Unsure	92 (15.57)	4 (11.43)	88 (15.83)	

a see Table 1, footnote 1 for other races.

b see Table 1, footnote 2 for list of other occupations.

c see Table 1, footnote 3 for list of comorbidities.

The bold values indicate the number is statistically significant (*P* ≤ .05)

### Home testing in June and November 2022

**Figure 1.**
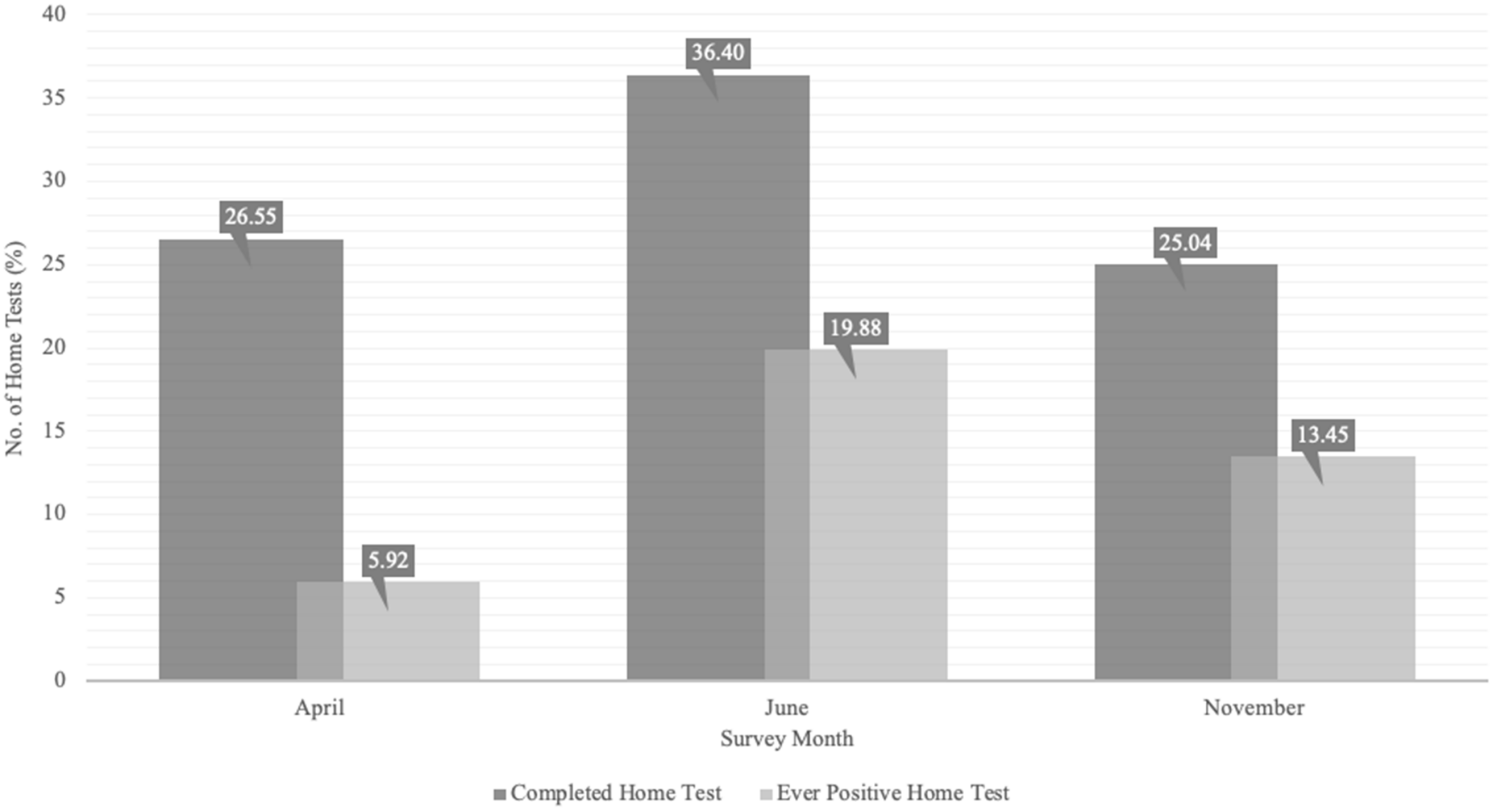
Completed home tests and ever-positive home tests by survey month.

Subsequent surveys were conducted in June and November, where home testing increased to 36.4% in June and decreased to 25% in November, compared to 26.6% in April (Figure 1). Of those who completed home tests in June and November, having at least one positive test increased from 5.9% to 19.9% in June and decreased to 13.5% in November (Figure 1). Demographics and factors associated with home testing or having at least one positive home test for COVID-19, were generally similar in both June and November, compared to April (Supplemental Tables 1 and 2).

## Discussion

Within Northwestern Medicine’s tertiary healthcare system, we found a significant association between completing a home test and perceived exposure to people, other than patients with known or suspected COVID-19 infection. In relation to the United States’ current national nursing workforce,^13^ our study sample revealed higher survey responses from nurses, compared to other occupational groups. Given the impact of reinfection rates among this workforce, having rapid antigen home tests available is crucial. A previous study comparing self-sampling home tests with professionally collected sampling found that self-sampling at home is a reliable alternative to professional site collected sampling.^14^ Given its reliability and accessibility, home tests could be additional tools of protection for this workforce. As previous studies reported, with an increase in COVID-19 prevalence, at-home testing also increased.^15^

Our study took place during the time that the predominant COVID-19 variant of concern was from the Omicron lineage in late 2021.^16^ Changes in pathogenicity and infectivity within the sub-lineages of Omicron resulted in higher transmissibility of viral infection.^17^ Despite this knowledge of emerging variants of concern, on February 28, 2022, the Chicago Department of Public Health lifted the mask and vaccine mandate requirements due to the decreased incidence in Chicago (7-day average of 1.5% test positivity and 283 cases per day).^18^ While severity was lower than the previously dominant Delta variant, the Omicron variants severely impacted the healthcare system, resulting in reinfection cases due to its high transmibility.^19^ Congruent with our study results, the increase in COVID-19 cases during July was on trend with the CDC nationally reported rise in COVID-19 infection cases during the summer of 2022.^20^ The decrease observed in-home testing in November, compared to April and July was also on trend with CDC data indicating that over 80% of U.S. counties had low COVID-19 community levels; which included Chicago and surrounding suburban counties.^21^ Signs of improvement in the matter of COVID-19 burden on communities was due to vaccination and active tracking, but further long-term improvements are needed to continue reducing the risk of mortality of COVID-19 infection as we entered the end of the public health emergency.^22^

With perceived exposure to COVID-19 infection being the main observed concern in our study that was associated with higher testing frequencies and ever positivity, it is important to create sustainable surveillance tools that are easily accessible to the entire U.S. population so that the public can stay abreast of COVID-19 cases. With community transmission levels often being the driving factor to establishing testing frequency protocols in high-risk settings,^23^ healthcare workplaces have the authority on how to incorporate testing at their sites. Surprisingly, the number of HCWs who reported being exposed to patients with known or suspected COVID-19 was relatively low. Given the use of personal protective equipment in healthcare settings, this correlates with studies that have found that personal protective equipment is a strong preventative strategy for communicable diseases, but alone does not completely reduce the risk of infection.^1,24^A published meta-analysis reported that a mixed prevention methods approach, including personal protective equipment, was the best-measured approach to controlling future COVID-19 outbreaks, both in healthcare settings and in the community.^5^

Our study has some important limitations to note. First, our data is representative of a single healthcare system in Chicago and surrounding suburban counties. Like many healthcare settings, PCR and rapid antigen testing were widely available and could have contributed to the low numbers of reported home testing compared to the citywide testing frequencies.^25^ Second, consistent with the gender and racial and ethnic characteristics of the U.S. healthcare workforce, our study was limited to predominately females identifying as non-Hispanic white race. Therefore, while this study is generalizable to this overall subset of HCWs in the U.S. population, this study may not be generalizable to other HCW populations across the U.S. Third, our survey data could be susceptible to recall bias, as HCWs were asked to recall on the past 30 days regarding their utilization of home tests. Unlike laboratory sampling, HCWs wouldn’t have laboratory records or appointment records of their date of testing and/or results. There is also the potential for false negatives in the home test when testing too early near infection.^26^ Thus, the reported number of home testing and positive tests could be underreported within this cohort, reducing the power of our analysis.

In summary, among this cohort of HCWs, we found that perceived communicable exposure was associated with higher home testing and ever-positive test frequencies. With the lack of published literature on the topic of self-administered COVID-19 home tests and given a published meta-analysis reporting a high pooled Omicron asymptomatic infection rate (32.4%),^27^ further studies are needed to prevent future cluster outbreaks of COVID-19 in healthcare settings. A mixed methods approach that includes valid, noninvasive, and quick self-administered home testing and reporting would be worth further studying in order to maneuver from exposure concerns being the determining factor that HCWs get tested and turn to a more sustainable preventative system of frequent testing to prevent asymptomatic transmission rates.

## Supporting information

Valdivia et al. supplementary material Valdivia et al. supplementary material

## Supplementary material

For supplementary material accompanying this paper visit http://doi.org/10.1017/ash.2024.17. click here to view supplementary material

## Thank you notes

We are grateful to the NM employees who volunteered to participate in this project.
