# Supplementary material for: Utilization of at-home tests for coronavirus disease 2019 (COVID-19) among healthcare workers in Chicago
Source: Antimicrob Steward Healthc Epidemiol. 2024 Apr 24;4(1):e58. doi: 10.1017/ash.2024.17 (PMC11062791; doi:10.1017/ash.2024.17)
Supplement: Valdivia et al. supplementary material [file S2732494X24000172sup001.docx]

**Supplemental Table 1.** Participant characteristics by completion of home testing for June and November 2022.

|  | June | | | | November | | | | |
| --- | --- | --- | --- | --- | --- | --- | --- | --- | --- |
|  | Overall,  n (%) | Completed home test,  n (%) | | *p*-value | Overall,  n (%) | | Completed home test,  n (%) | | *p*-value |
| Characteristic |  | **Yes** | **No** |  |  | **Yes** | | **No** |  |
| n (%) | 1,783 (100.00) | 649 (36.40) | 1,134 (63.60) |  | 1,426 (100.00) | 357 (25.04) | | 1,069 (74.96) |  |
| Age |  |  |  | **<.001** |  |  | |  | 0.12 |
| 18-29 | 213 (11.95) | 90 (13.87) | 123 (10.85) |  | 151 (10.59) | 44 (12.32) | | 107 (10.01) |  |
| 30-39 | 534 (29.95) | 229 (35.29) | 305 (26.90) |  | 414 (29.03) | 110 (30.81) | | 304 (28.44) |  |
| 40-49 | 438 (24.57) | 163 (25.12) | 275 (24.25) |  | 348 (24.40) | 93 (26.05) | | 255 (23.85) |  |
| $\boldsymbol{\geq}$50 | 598 (24.57) | 167 (25.73) | 431 (38.01) |  | 513 (35.97) | 110 (30.81) | | 403 (37.70) |  |
| Sex |  |  |  | 0.11 |  |  | |  | 0.49 |
| Female | 1,463 (82.05) | 520 (80.12) | 943 (83.16) |  | 1,169 (81.98) | 297 (83.19) | | 872 (81.57) |  |
| Male | 320 (17.95) | 129 (19.88) | 191 (16.84) |  | 257 (18.02) | 60 (16.81) | | 197 (18.43) |  |
| Race/Ethnicity |  |  |  | 0.10 |  |  | |  | 0.71 |
| Asian | 148 (8.30) | 62 (9.55) | 86 (7.58) |  | 105 (7.36) | 27 (7.56) | | 78 (7.30) |  |
| Hispanic/Latino | 96 (5.38) | 27 (4.16) | 69 (6.08) |  | 79 (5.54) | 17 (4.76) | | 62 (5.80) |  |
| Non-Hispanic Black | 42 (2.36) | 12 (1.85) | 30 (2.65) |  | 37 (2.59) | 7 (1.96) | | 30 (2.81) |  |
| Non-Hispanic White | 1,459 (81.83) | 538 (82.90) | 921 (81.22) |  | 1,173 (82.26) | 300 (84.03) | | 873 (81.67) |  |
| Other/Prefer not to answer | 38 (2.13) | 10 (1.54) | 28 (2.47) |  | 32 (2.24) | 6 (1.68) | | 26 (2.43) |  |
| Occupation |  |  |  | **<.001** |  |  | |  | **0.02** |
| Administrative role | 262 (14.69) | 83 (12.79) | 179 (15.78) |  | 226 (15.85) | 54 (15.13) | | 172 (16.09) |  |
| Nurse (Practitioner, Registered, or Equivalent) | 490 (27.48) | 167 (25.73) | 323 (28.48) |  | 381 (26.72) | 108 (30.25) | | 273 (25.54) |  |
| Physician | 337 (18.90) | 169 (26.04) | 168 (14.81) |  | 254 (17.81) | 75 (21.01) | | 179 (16.74) |  |
| Other | 694 (38.92) | 230 (35.44) | 464 (40.92) |  | 565 (39.62) | 120 (33.61) | | 445 (41.63) |  |
| Comorbidities |  |  |  | **0.02** |  |  | |  | 0.24 |
| No | 1,209 (67.81) | 462 (71.19) | 747 (65.87) |  | 946 (66.34) | 246 (68.91) | | 700 (65.48) |  |
| Yes | 574 (32.19) | 187 (28.81) | 387 (34.13) |  | 480 (33.66) | 111 (31.09) | | 369 (34.52) |  |
| I was exposed to people, other than patients with known or suspected COVID-19 |  |  |  | **<.001** |  |  | |  | **<.001** |
| No | 651 (36.51) | 160 (24.65) | 491 (43.30) |  | 653 (45.79) | 110 (30.81) | | 543 (50.80) |  |
| Yes | 709 (39.76) | 379 (58.40) | 330 (29.10) |  | 368 (25.81) | 160 (44.82) | | 208 (19.46) |  |
| Unsure | 423 (23.72) | 110 (16.95) | 313 (27.60) |  | 405 (28.40) | 87 (24.37) | | 318 (29.75) |  |
| I was exposed to patients with known or suspected COVID-19 |  |  |  | 0.08 |  |  | |  | 0.93 |
| No | 775 (43.47) | 265 (40.83) | 510 (44.97) |  | 661 (46.35) | 166 (46.50) | | 495 (46.30) |  |
| Yes | 705 (39.54) | 279 (42.99) | 426 (37.57) |  | 512 (35.90) | 130 (36.41) | | 382 (35.73) |  |
| Unsure | 303 (16.99) | 105 (16.18) | 198 (17.76) |  | 253 (17.74) | 61 (17.09) | | 192 (17.96) |  |

**Supplemental Table 2.** Participant characteristics by ever positive home test for June and November 2022.

|  | June | | | | November | | | |
| --- | --- | --- | --- | --- | --- | --- | --- | --- |
|  | Overall, n (%) | Ever Positive, n (%) | | *p*-value | Overall, n (%) | Ever Positive, n (%) | | *p*-value |
| Characteristic |  | **Yes** | **No** |  |  | **Yes** | **No** |  |
| n (%) | 649 (100.00) | 129 (19.88) | 520 (80.12) |  | 357 (100.00) | 48 (13.45) | 309 (86.55) |  |
| mean (SD) | 2.29 (1.68) | 1.67 (1.16) |  |  | 2.07 (1.39) | 1.56 (0.82) |  |  |
| Age |  |  |  | **0.01** |  |  |  | 0.15 |
| 18-29 | 90 (13.87) | 7 (5.43) | 83 (15.96) |  | 44 (12.32) | 3 (6.25) | 41 (13.27) |  |
| 30-39 | 229 (35.29) | 46 (35.66) | 183 (35.19) |  | 110 (30.81) | 14 (29.17) | 96 (31.07) |  |
| 40-49 | 163 (25.12) | 36 (27.91) | 127 (24.42) |  | 93 (26.05) | 10 (20.83) | 83 (26.86) |  |
| $\boldsymbol{\geq}$50 | 167 (25.73) | 40 (31.01) | 127 (24.42) |  | 110 (30.81) | 21 (43.75) | 89 (28.80) |  |
| Sex |  |  |  | 0.12 |  |  |  | **0.01** |
| Female | 520 (80.12) | 97 (75.19) | 423 (81.35) |  | 297 (83.19) | 34 (70.83) | 263 (85.11) |  |
| Male | 129 (19.88) | 32 (24.81) | 97 (18.65) |  | 60 (16.81) | 14 (29.17) | 46 (14.89) |  |
| Race/Ethnicity |  |  |  | 0.79 |  |  |  | 0.70 |
| Asian | 62 (9.55) | 13 (10.08) | 49 (9.42) |  | 27 (7.56) | 3 (6.25) | 24 (7.77) |  |
| Hispanic/Latino | 27 (4.16) | 7 (5.43) | 20 (3.85) |  | 17 (4.76) | 2 (4.17) | 15 (4.85) |  |
| Non-Hispanic Black | 12 (1.85) | 1 (0.78) | 11 (2.12) |  | 7 (1.96) | 1 (2.08) | 6 (1.94) |  |
| Non-Hispanic White | 538 (82.90) | 106 (82.17) | 432 (83.08) |  | 300 (84.03) | 40 (83.33) | 260 (84.14) |  |
| Other/Prefer not to answer | 10 (1.54) | 2 (1.55) | 8 (1.54) |  | 6 (1.68) | 2 (4.17) | 4 (1.29) |  |
| Occupation |  |  |  | 0.22 |  |  |  | 0.79 |
| Administrative role | 83 (12.79) | 18 (13.95) | 65 (12.50) |  | 54 (15.13) | 9 (18.75) | 45 (14.56) |  |
| Nurse (Practitioner, Registered, or Equivalent) | 167 (25.73) | 28 (21.71) | 139 (26.73) |  | 108 (30.25) | 12 (25.00) | 96 (31.07) |  |
| Physician | 169 (26.04) | 42 (32.56) | 127 (24.42) |  | 75 (21.01) | 10 (20.83) | 65 (21.04) |  |
| Other | 230 (35.44) | 41 (31.78) | 189 (36.35) |  | 120 (33.61) | 17 (35.42) | 103 (33.33) |  |
| Comorbidities |  |  |  | 0.97 |  |  |  | 0.52 |
| No | 462 (71.19) | 92 (71.32) | 370 (71.15) |  | 246 (68.91) | 35 (72.92) | 211 (68.28) |  |
| Yes | 187 (28.81) | 37 (28.68) | 150 (28.85) |  | 111 (31.09) | 13 (27.08) | 98 (31.72) |  |
| I was exposed to people, other than patients with known or suspected COVID-19 |  |  |  | **<.001** |  |  |  | **<.001** |
| No | 160 (24.65) | 11 (8.53) | 149 (28.65) |  | 110 (30.81) | 6 (12.50) | 104 (33.66) |  |
| Yes | 379 (58.40) | 100 (77.52) | 279 (53.65) |  | 160 (44.82) | 36 (75.00) | 124 (40.13) |  |
| Unsure | 110 (16.95) | 18 (13.95) | 92 (17.69) |  | 87 (24.37) | 6 (12.50) | 81 (26.21) |  |
| I was exposed to patients with known or suspected COVID-19 |  |  |  | 0.09 |  |  |  | **0.04** |
| No | 265 (40.83) | 44 (34.11) | 221 (42.50) |  | 166 (46.50) | 29 (60.42) | 137 (44.34) |  |
| Yes | 279 (42.99) | 57 (44.19) | 222 (42.69) |  | 130 (36.41) | 16 (33.33) | 114 (36.89) |  |
| Unsure | 105 (16.18) | 28 (21.71) | 77 (14.81) |  | 61 (17.09) | 3 (6.25) | 58 (18.77) |  |
